# Supplementary material for: The Diagnostic Accuracy of Magnetic Resonance Imaging for Maternal Acute Adnexal Torsion during Pregnancy: Single-Institution Clinical Performance Review
Source: J Clin Med. 2020 Jul 13;9(7):2209. doi: 10.3390/jcm9072209 (PMC7408813; doi:10.3390/jcm9072209)
Supplement: Supplementary file 1 [file jcm-09-02209-s001.pdf]

Supplemental materials

Supplemental Table S1. MRI features of ovarian torsion

(a) Tubal wall thickness (>10 mm)
(b) Whirlpool sign (a twisted ovarian pedicle or twisted fallopian tube)
(c) Peritoneal fluid
(d) Uterine deviation to the twisted side
(e) Periadenxal fat stranding
(f) Ovarian stromal (medullary) edema
(g) Symmetrical or asymmetrical ovarian cystic wall thickness
(h) Prominent follicles in the periphery of the ovarian parenchyma (4 mm or more)
(i) ADC values in the ovarian medulla
(k) ADC values in ovarian cortex

Supplemental Table S2. Correlation between magnetic resonance image and surgical pathological diagnosis

Magnetic resonance image	Final diagnostic surgical outcome with/without pathology results			
	Adnexal torsion	No adnexal torsion	No surgical evaluation	Total
Adnexal torsion	19	2	3	24
No adnexal torsion	0	7	6	13
Total	19	9	9	37

Ultrasonography image	Final diagnostic surgical outcome with/without pathology results			
	Adnexal torsion	No adnexal torsion	No surgical evaluation	Total
Visualized ovary on USG (n = 28 †)				
Adnexal torsion diagnosis: (Grayscale +/- Doppler studies among visualized ovary on sonography)	10	1	1	12
No adnexal torsion diagnosis: (Normal doppler flow among visualized ovary on sonography)	6	5	5	16
Non-visualized ovary on USG (n = 9)	3	3	3	9
Total	19	9	9	37

Note. † 5 women did not undergo Doppler studies, USG, Ultrasonography image.

Supplemental Table S3. Rates of adnexal torsion diagnosed by MRI after ultrasound visualization of the ovary

MRI findings	Diagnostic ultrasonography,n (%)	
	Diagnostic adnexal torsion (n = 28)	No diagnostic adnexal torsion (n = 9 ‡)
Adnexal torsion	20(71.4%)	4†(44.4%)
No adnexal torsion	8(28.6%)	5(65.6%)

† Three women were identified as having adnexal torsion on diagnostic surgery (18, 21.6, and 31.6 weeks of gestational age, respectively). One woman did not undergo surgery due to symptom relief through USG-guided aspiration of an ovarian cyst at 30.5 weeks' gestation.‡ In women with nondiagnostic USG, non-surgical women (n = 3; 28, 30.5, and 32.2 weeks of gestational age) due to symptom relief, adnexal torsion women (n = 3; 18, 21.6, and 31.6 weeks of gestational age), and non-adnexal torsion women (n = 3; 29.6, 30.1, and 36.3 weeks of gestational age) through diagnostic surgery have consisted in the 2nd and 3rd trimesters.

Supplemental Table S4. Inter-observer agreement for the different MR features by surgical methods

MRI torsion features	Cystectomy and Detorsion Agreement between Readers 1 and 2	*P*-value	Salpingo-oophorectomy Agreement between Readers 1 and 2	*P*-value
Whirlpool sign (a twisted ovarian pedicle or twisted fallopian tube)	1(1.000–1.000)	0.008	1(1.000–1.000)	0.008-
Tubal wall thickness	1(1.000–1.000)	0.008	1(1.000–1.000)	0.008
Symmetrical or asymmetrical ovarian cystic wall thickness	1(1.000–1.000)	0.008	1(1.000–1.000)	0.008
Ovarian stromal(medullary) edema	1(1.000–1.000)	0.008	1(1.000–1.000)	0.008
Prominent follicles in in the periphery of the ovarian parenchyma(4mm or more)	0.500 (0.076–0.924)	0.046	1.000(1.000–1.000)	0.008
Periadenexal fat stranding	0.625 (0.027–1.277)	0.020	0.696(0.170–1.222)	0.053
Uterine deviation to the twisted side	0.625 (0.027–1.277)	0.020	0.588(0.093–1.269)	0.088
Peritoneal fluid	0.636 (0.208–1.065)	0.018	0.696(0.170–1.222)	0.053
